# Mucormycosis: Literature review and retrospective report of 15 cases from Portugal

**DOI:** 10.18502/cmm.6.4.5437

**Published:** 2020-12

**Authors:** Beatriz Prista Leão, Isabel Abreu, Ana Cláudia Carvalho, António Sarmento, Lurdes Santos

**Affiliations:** 1 Infectious Diseases Department, Centro Hospitalar Universitário de São João, Oporto, Portugal; a Both authors contributed equally to this manuscript as joint first authors

**Keywords:** Immunosuppression, Invasive fungal infections, Mucormycosis

## Abstract

**Background and Purpose:**

Prevalence of mucormycosis is growing with the increase of the population at risk. Current recommendations for its management are mostly based on retrospective studies. 3 study aimed to present the cumulative experience of an Infectious Diseases Department from a Portuguese hospital in the management of mucormycosis and discuss the potential gaps in the diagnostic and therapeutic approaches of this infection.

**Materials and Methods:**

For the purposes of the study, the electronic hospital database was searched for adult patients with mucormycosis from 1996 to 2019 based on the definition provided by the Consensus Definitions of Invasive Fungal Disease. Demographic, clinical, treatment, and outcome data were collected and compared to what had been described in the related literature.

**Results:**

In total, 15 cases of mucormycosis were found, including 11 cases with sinus involvement (10 with central nervous system involvement), two pulmonary, and two gastrointestinal infections. Diabetes mellitus (n=7) and corticosteroid therapy (n=7) were frequent risk factors. Median duration of symptoms before the suspicion of diagnosis was 26 days (3-158). The diagnosis was confirmed in 12 patients mostly by histopathology (n=9); the culture was positive only once. Systemic antifungals and surgical debridement were the backbones of treatment; however, side effects, the need for therapeutic drug monitoring, and the anatomical location of lesions added complexity to management. Overall, seven patients died, two of them before the consideration of clinical suspicion.

**Conclusion:**

More medications are becoming available for the treatment of mucormycosis. Nevertheless, we believe that its prognosis will only significantly change through the increase of awareness and reduction of the time to diagnosis. An effective multidisciplinary approach among surgeons, infectious diseases specialists, radiologists, microbiologists, and anatomopathologists is critical to the achievement of this goal.

## Introduction

Mucormycosis is a rare invasive fungal disease responsible for significant morbidity and mortality in immunocompromised patients, especially those with haematological malignancies, hematopoietic stem cell transplant recipients, and patients who undergo chronic high-dose corticosteroid treatment [ 1-9 ]. Poorly controlled diabetes mellitus (DM) is also a major risk factor and frequently the most reported underlying condition of this infection [ 1 , 3-10 ]. 

It should also be noted that despite its rarity, it has been increasingly reported in recent years [ 4 , 8-10 ]. Improvement of the supportive care of immune-compromised patients with consequent higher survival rates, the use of effective antifungal agents against Candida spp or Aspergillus spp, new immunosuppressive therapies (e.g., chemotherapy or targeted cancer immunotherapy), higher awareness of this infection, and the availability of better diagnostic tools might have contributed to the rising incidence of invasive mucormycosis in the aforementioned at-risk patients [ 2-4 , 11 ]. 

Overall prognosis of mucormycosis remains remarkably poor, with an associated mortality rate of up to 80%, depending on the underlying conditions and presentation [ 1 , 4 , 6 , 10 , 11 ]. Delays in the clinical recognition of infection together with its aggressive nature and difficult management are important factors for the reported poor outcomes [ 4 , 5 , 12-14 ]. However, the net state of immunosuppression of the host, the poor prognosis of some of the underlying diseases, the fact that urgent treatments often need to be postponed due to the infection, and also events of decompensation of comorbidities are also relevant contributors [ 4 , 5 , 12-14 ]. 

The recommended approach to invasive mucormycosis is mostly based on retrospective studies [ 2 , 4 , 11 ]. Both antifungal and surgical management are recognized as the backbones of the treatment; however, both pose challenging issues, such as medication toxicities and anatomic location of lesions [ 1 , 3 , 4 , 7 , 11 , 14 , 15 ]. 

This study aimed to present the cumulative experience of a Portuguese tertiary care center regarding the management of patients with invasive mucormycosis and discuss the potential gaps in the diagnosis and therapeutic approaches while sharing an approach suggestion. 

## Materials and Methods

For the purposes of the research, a retrospective observational cohort study was performed at the Infectious Diseases (ID) Department of a tertiary care center in Oporto, Portugal. Electronic hospital database was reviewed for cases of mucormycosis and all adult patients (18 years) admitted until April 2019 were included (the first case was reported in December 1996). Moreover, the clinical records were used to obtain the demographic and clinical data of the patients. 

The disease was defined based on the infection site, including sinus or rhino-orbital-cerebral mucormycosis (SM), pulmonary mucormycosis (PM), gastrointestinal mucormycosis (GM), cutaneous (CM), and renal mucormycosis (RM). Moreover, the central nervous system (CNS) involvement was considered if there was an intracranial extension, cavernous sinus thrombosis, or internal carotid infarction. The disseminated disease was considered if two or more non-contiguous sites were involved. 

Diagnostic criteria were based on the Consensus Definitions of Invasive Fungal Disease [ 16 ]. Probable invasive mucormycosis (IM) was considered in patients with predisposing risk factors, compatible clinical and radiological features, and improvement with systemic antifungals. The diagnosis was confirmed by Mucorales growth on cultures, histopathological examination revealing fungal structures compatible with Mucorales, or positive amplification of Mucorales DNA by polymerase-chain-reaction (PCR). 

Therapeutic regimens were analyzed for antifungals, surgical debridement, and adjunctive therapies (i.e., hyperbaric oxygen therapy and granulocyte colony-stimulating factors). Evaluated clinical outcomes were survival or death (overall and attributable mortality). 

**Ethical considerations**

This study was conducted in accordance with the Declaration of Helsinki and approved by the Ethics Committee of the institution that waived the informed consent of the patients. 

## Results

In total, 15 IM cases were identified, 11 of whom had been diagnosed over the last 10 years (2010-2019). Median age of the patients was 53 years (interquartile range : 22) and 10 out of 15 cases were male. Table 1 summarizes the detailed information on each case. 

**Table 1:** Detailed information about mucormycosis cases diagnosed at the Infectious Diseases Department of the University Hospital Center of São João, Portugal during 1996-2019

Gender, age (in years); Predisposing condition (s)	Type of disease (year of diagnosis)	Presenting symptoms	Imaging results	Etiological diagnosis: technique (s) and sample (s)	Duration of disease (days)	Treatment: Antifungals (duration; associated toxicity) Surgery (N) Adjuvant therapies	Outcome and	Sequelae
Male, 22; IVDU	Rhino-orbital-cerebral mucormycosis (1996)	Fever; headache; ophthalmoplegia; cranial nerve palsies; altered state of consciousness	Brain CT image: focal cerebral lesions	Histopathology (brain necropsy sample): suggestive Mucorales structures (1/1) Culture: N/P PCR: N/P	Symptoms - suspicion: 26 Symptoms - diagnosis: 26 Suspicion - treatment: N/A Suspicion - diagnosis: N/A Symptoms - death: 26	None (diagnosis made on necropsy specimen) Antifungals: N/P Surgery: 0 Adjuvant therapies: N/P	Death, directly attributed to mucormycosis
Female, 53; Type 2 DM	Rhino-orbital-cerebral mucormycosis (2001)	Nasal mucosa ulceration; periorbital cellulitis, ophthalmoplegia; visual impairment; cranial nerve palsies; altered state of consciousness	Brain CT: sinusitis, focal cerebral lesions.	Histopathology (nasal ulcer): suggestive Mucorales structures (1/1) Culture: negative (1/1) PCR: N/P	Symptoms - suspicion: 3 Symptoms - diagnosis: 6 Suspicion - treatment: 0 Suspicion - diagnosis: 3 Symptoms - death: 21	Antifungals (18 days): L-AMB Surgery: 0 Adjuvant therapies: N/P	Death, directly attributed to mucormycosis
Male, 78; Type 2 DM, myelodysplastic syndrome, corticosteroid therapy	Pulmonary mucormycosis (2004)	Fever; Hemoptysis	Thoracic CT: pulmonary cavity involving more than one lobe; pulmonary nodules	Histopathology (BAL): negative (1/1) Culture (BAL and sputum): *Rhizopus spp* (1/6) PCR: N/P	Symptoms - suspicion: 24 Symptoms - diagnosis: 30 Suspicion - treatment: 0 Suspicion - diagnosis: 6 Symptoms - death: 29	Antifungals (5 days): AMB-d Surgery: 0 Adjuvant therapies: N/P	Death, directly attributed to mucormycosis
Male, 42; HIV, IVDU	Gastrointestinal mucormycosis (2005)	Abdominal pain; nausea/vomiting; dysphagia; constipation; weight loss; septic shock.	Abdominal CT: splenic abscesses, colitis with colonic distension, free intraperitoneal fluid	Histopathology (colic necropsy samples): suggestive Mucorales structures (1/1) Culture: N/P PCR: N/P	Symptoms - suspicion: N/A Symptoms - diagnosis: 37 Suspicion - treatment: N/A Suspicion - diagnosis: N/A Symptoms - death: 37	None (diagnosis made on necropsy specimen) Antifungals: N/P Surgery: 0 Adjuvant therapies: N/P	Death, directly attributed to mucormycosis
Male, 49; Reiter's syndrome, sulfasalazine and corticosteroids therapy, with secondary neutropenia	Gastrointestinal mucormycosis (2011)	Abdominal pain; nausea/vomiting; septic shock	Abdominal CT: diffuse parietal thickening of the descending and transverse colon	Histopathology (colonic ulcers): suggestive Mucorales and *Candida spp* structures (1/1) Culture: *Candida albicans* (1/1) PCR: N/P	Symptoms - suspicion: 17 Symptoms - diagnosis: 17 Suspicion - treatment: 0 Suspicion - diagnosis: N/A Symptoms - death: N/A	Antifungals (173 days): AMB-d, followed by posaconazole Surgery: 0 Adjuvant therapies: N/P	Survived; No relevant sequel
Female, 39; Type 2 DM, corticosteroid therapy for cutaneous porphyria	Rhino-orbital-cerebral mucormycosis (2013)	Sinusitis; headache; periorbital edema; visual impairment; ophthalmoplegia; nausea/vomiting	Brain CT: sinusitis, cavernous sinus involvement/ thrombosis, cerebritis	Histopathology (periorbital, sinus): suggestive Mucorales structures (2/3) Culture: *C. albicans* (1/3), negative (2/3) PCR: N/P	Symptoms - suspicion: 5 Symptoms - diagnosis: 15 Suspicion - treatment: 0 Suspicion - diagnosis: 10 Symptoms - death: 727	Antifungals (267 days): L-AMB (nausea/vomiting) Surgery (2):debridement; exenteration Adjuvant therapies: HBO	Unilateral orbital exenteration; Death from a cardiovascular event, unrelated to mucormycosis
Male, 55; Type 2 DM, Idiopathic TCD4+ lymphopenia, corticosteroid therapy	Rhino-orbital-cerebral mucormycosis (2013)	Sinusitis; headache; nasal mucosa ulceration; facial and periorbital edema; visual impairment; ophthalmoplegia and other cranial nerve palsies	Brain CT: sinusitis, cerebritis. Brain MRI: nodular irregular cerebellar lesion; sinusitis	Histopathology (palate): suggestive Mucorales structures (2/2) Culture: N/P PCR: N/P	Symptoms - suspicion: 157 Symptoms - diagnosis: 157 Suspicion - treatment: 0 Suspicion - diagnosis: 0 Symptoms - death: 176	Antifungals (19 days): AMB-d (AKI*), followed by L-AMB + caspofungin Surgery (1): debridement Adjuvant therapies: N/P	Death, directly attributed to mucormycosis
Female, 56; Rheumatoid arthritis, corticosteroid therapy	Rhino-orbital-cerebral mucormycosis (2015)	Headache; proptosis; visual impairment; ophthalmoplegia and other cranial nerve palsies	Brain CT: cavernous sinus involvement/ thrombosis	None (presumptive) Histopathology: N/P Culture: N/P PCR: N/P	Symptoms - suspicion: 33 Symptoms - diagnosis: N/A Suspicion - treatment: 1 Suspicion - diagnosis: N/A Symptoms - death: N/A	Antifungals (503 days): L-AMB, followed by posaconazole Surgery: N/P Adjuvant therapies: HBO	Survived; No relevant sequel
Male, 69; Type 2 DM	Rhino-orbital-cerebral mucormycosis (2015)	Fever; cranial nerve palsies; altered state of consciousness	Brain CT and MRI: sinusitis, an infiltrative lesion in the right posterolateral aspect of the nasopharynx	None (presumptive) Histopathology (sinus): negative (inflammation and necrosis; 8/8) Culture: *Staphylococcus aureus* (2/5); *C. albicans* (1/5); *Streptococcus pneumoniae* (1/5); negative (3/5) PCR: N/P	Symptoms - suspicion: 31 Symptoms - diagnosis: N/A Suspicion - treatment: 0 Suspicion - diagnosis: N/A Symptoms - death: N/A	Antifungals (522 days): L-AMB, followed by posaconazole Surgery (4): debridement Adjuvant therapies: N/P	Survived; No relevant sequel
Female, 83;	Isolated sinus mucormycosis (2016)	Sinusitis	Brain CT: sinusitis	Histopathology (sinus): suggestive Mucorales structures (1/1) Culture: negative (1/1) PCR: N/P	Symptoms - suspicion: 91 Symptoms - diagnosis: 91 Suspicion - treatment: 66** Suspicion - diagnosis: 0 Symptoms - death: N/A	Antifungals (510 days): L-AMB, followed by posaconazole Surgery (1): debridement Adjuvant therapies: HBO	Survived; No relevant sequel
Male, 55; Type 2 DM, cirrhosis, history of hepatocellular carcinoma (resected in 2012)	Rhino-orbital-cerebral mucormycosis (2017)	Sinusitis; headache; diplopia; VI cranial nerve palsy	Brain CT and MRI: sinusitis, cavernous sinus involvement/ thrombosis; focal, nodular, irregular cerebral lesions; cerebritis	Histopathology (sinus): negative (2/2) Culture: *Enterococcus faecalis* (1/2), negative (1/2) PCR: DNA Zygomycetes (2/2)	Symptoms - suspicion: 8 Symptoms - diagnosis: 16 Suspicion - treatment: 0 Suspicion - diagnosis: 8 Symptoms - death: N/A	Antifungals (197 days): L-AMB, followed by posaconazole Surgery (1): debridement Adjuvant therapies: N/P	Survived; No relevant sequel
Male, 76; Small cell lymphocytic lymphoma, corticosteroid therapy	Rhino-orbital-cerebral mucormycosis (2017)	Sinusitis; ocular pain; proptosis; visual impairment; ophthalmoplegia	Brain CT and MRI: sinusitis, cavernous sinus involvement/ thrombosis	Histopathology (sinus): suggestive Mucorales structures (1/3) Culture: *S. aureus* (1/3), *Corynebacterium tuberculostearicum* (1/3), *Aspergillus fumigatus* (1/3) ***, negative (2/3) PCR: *A. fumigatus* DNA (1/2), negative (1/2)	Symptoms - suspicion: 14 Symptoms - diagnosis: 50 Suspicion - treatment: 0 Suspicion - diagnosis: 36 Symptoms - death: N/A	Antifungals (920 days): L-AMB (AKI), followed by posaconazole Surgery (1): debridement Adjuvant therapies: HBO	Survived; Sequelar amaurosis of left eye
Female, 21; Type 1 DM	Rhino-orbital-cerebral mucormycosis (2018)	Sinusitis; periorbital edema; ocular pain; proptosis; visual impairment; ophthalmoplegia and other cranial nerve palsies; diabetic ketoacidosis	Brain CT: sinusitis, right internal carotid artery, and cavernous sinus involvement	Histopathology (sinus, orbital): suggestive Mucorales structures (1/4) Culture: *Klebsiella oxytoca* (1/7), *E. faecalis* (1/7), *S. aureus* (2/7), *C. albicans* (1/7), *Serratia marcescens* (1/7), *Staphylococcus epidermidis* (1/7), negative (4/7) PCR: Mucorales DNA (1/2)	Symptoms - suspicion: 48 Symptoms - diagnosis: 75 Suspicion - treatment: 0 Suspicion - diagnosis: 27 Symptoms - death: N/A	Antifungals (518 days): L-AMB (AKI*), followed by posaconazole (uncoercive nausea/vomiting*), followed by isavuconazole Surgery (4): debridement, exenteration Adjuvant therapies: HBO	Survived (still under antifungal therapy); Unilateral orbital exenteration
Male, 38; Type 1 DM	Rhino-orbital-cerebral mucormycosis (2019)	Sinusitis; headache; periorbital edema; ocular pain; proptosis; ophthalmoplegia; visual impairment	Brain MRI: left cavernous sinus involvement/ thrombosis; lesion in the orbital apex, with infiltration and thickening of the left eye muscles and optic nerve	None (presumptive) Histopathology (sinus and exenteration specimen): negative (3/3) Culture: *S. epidermidis* (3/3), *Proteus mirabilis* (1/3) PCR: negative (3/3)	Symptoms - suspicion: 158 Symptoms - diagnosis: N/A Suspicion - treatment: 0 Suspicion - diagnosis: N/A Symptoms - death: N/A	Antifungals (419 days): L-AMB (AKI*), followed by isavuconazole (phlebitis), followed by posaconazole Surgery (3): debridement, exenteration Adjuvant therapies: HBO	Survived; Unilateral orbital exenteration
Male, 43; Myasthenia gravis, corticosteroid therapy, iatrogenic DM	Pulmonary mucormycosis (2019)	Fever; hemoptysis; ketoacidosis	Thoracic CT: cavitated pulmonary lesion with a left hilar mass effect	Histopathology (pulmonary tissue): negative (1/1) Culture: negative (1/1) PCR: Mucorales DNA (1/1)	Symptoms - suspicion: 25 Symptoms - diagnosis: 25 Suspicion - treatment: 0 Suspicion - diagnosis: 0 Symptoms - death: 47	Antifungals (21 days): L-AMB Surgery: 0 Adjuvant therapies: N/P	Death, directly attributed to mucormycosis

N: number; IVDU: intravenous drug use; CT: computed tomography scan; MRI: magnetic resonance imaging; N/A: not applicable; N/P: not performed; PCR: polymerase chain reaction; DM: Diabetes mellitus; HIV: Human Immunodeficiency Virus; HBO: hyperbaric oxygen; AMB: Amphotericin B; L-AMB: liposomal amphotericin B; AMB-d: amphotericin B deoxycholate; AKI: acute kidney injury; BAL: bronchoalveolar lavage

*Toxicity requiring dose reduction or discontinuation of the medicine.

**Patient initially was managed in another hospital and was started on L-AMB upon admission in our institution.

***Considering the findings of histopathology and the negativity of the remaining samples for this agent (either in culture or molecular biology), the identification of Aspergillus in these samples was interpreted as contamination.

Both DM (n=8) and corticosteroid treatment (n=7) were frequent risk-factors. The SM was the most common presentation (n=11), mostly with CNS involvement (n=10); sinusitis and unilateral ophthalmoplegia were the most frequent complaints (n=8) followed by headache, visual loss, and other cranial nerve palsies (n=7). 

Overall, 6 out of the 15 studied patients required ICU admission for reasons other than post-surgical management: septic shock (n=3), neurological deterioration (n=1), ketoacidosis (n=1), and respiratory failure (n=1). The IM was confirmed in 12 patients, and the culture was positive only once. Suggestive histopathological findings were found in nine patients while in eight of them, it was the only evidence for the diagnosis. The PCR was performed on five patients and was positive in three of them. 

Patients reported symptoms for a median of 26 days (IQR: 25, range: 3-158) before mucormycosis was suspected. Amphotericin B (AMB) was administered to all patients as soon as the hypothesis of mucormycosis was raised except for one patient who was transferred from another hospital. The antifungals were administered to this patient only when admitted to our institution which was 66 days after clinical suspicion. It must be noted that AMB nephrotoxicity was the most common side effect. Whenever a switch to oral therapy was possible, posaconazole was the most used medication (seven out of eight cases). 

In total, eight, four, and three patients underwent surgical debridement, needed multiple procedures, and required radical surgery with orbital exenteration, respectively. It should be mentioned that five patients received solely antifungal therapy, two of whom had SM, PM, and multifocal extensive lesions; therefore, no benefit was expected from surgery. One of them who had PM died the day before surgery due to massive hemoptysis and another patient with SM had a prohibitive surgical risk; moreover, one GM patient was not considered for surgery. It must be mentioned that only the last two patients survived. 

In patients who survived the first month of treatment (n=9), the median duration of antifungals was 16.8 months (IQR: 8.4, range: 5.8-30.7). It must also be noted that seven patients died (47%), six of them directly due to mucormycosis, and two of them before the suspicion of the diagnosis (mucormycosis was diagnosed on necropsy). 

## Discussion

Rarity of invasive mucormycosis has limited the generalization of epidemiological findings based on the related literature [ 2 , 5-7 , 9 ]. Additionally, important geographical variations (with the possible influence of socioeconomic factors and occurrence of natural disasters) also have contributed to significant differences in the found clinical syndromes and predisposing conditions [ 2 , 5-10 ]. In fact, while DM is the most frequent risk factor for IM in low- and middle-income countries, hematological malignancy and transplantation are the major underlying conditions of IM patients in the western world [ 8-10 ]. 

The DM was consistently reported as an important risk factor in previous studies as was the case in the present research as well [ 1 , 3-8 , 10 , 14 ]. Despite the fact that stricter glucose control is not a proven protective factor, ketoacidosis is clearly associated with IM, particularly that caused by Rhizopus species, and occurred in two out of the nine diabetic patients [ 1 , 5-8 , 17 , 18 ]. Rhizopus spp., the most frequent etiologic agents of mucormycosis, are more often associated with SM, which was the most common presentation of IM in our series [ 8 , 9 ]. Other species seem to be more often related to different clinical presentations, such as PM or disseminated disease in the case of Cunninghamella spp. or CM in cases of Apophysomyces and Saksenaea spp. infection [ 8 , 9 ]. 

Corticosteroids were also frequent (n=7); therefore, conditions less commonly associated with mucor-mycosis, such as autoimmune diseases, should not be disregarded and corticosteroid use should be actively sought. 

The reported increase in mucormycosis incidence over the last decades was also evident in our series since a higher than three-fold increase occurred between the 2000s and the 2010s [ 2 , 7 , 18 , 19 ]. Greater awareness of IM, proactive and earlier diagnostic investigation, and enhanced diagnostic capability are probably important contributors. Despite the fact that it was frequently reported in previous research, only four patients had a fever in this study which emphasizes that its absence should not delay investigation [ 4 , 20 , 21 ]. 

Intracranial involvement is reported in up to 55% of SM cases [ 12 , 22 , 23 ]. In this series, 10 out of 11 patients had CNS disease possibly due to a considerable delay in clinical suspicion. In addition, 5 out of 10 patients had a concurrent bacterial infection which may have contributed as a facilitating factor for intracranial fungal dissemination. 

Bacterial coinfection is often present and, while antibacterial treatment is advisable, we warn against stopping antifungals whenever the clinical and imaging findings support mucormycosis, even if preliminary microbiological data is negative for fungi. In the present series, multiple invasive samples for microbiology and histopathology were often required before a diagnosis was made; meanwhile, only bacteria were isolated in biopsy specimens. In these patients, antibacterial treatment was prescribed; however, antifungals were consistently maintained. 

Diagnosis of mucormycosis is challenging and relies on a combination of a suggestive clinical picture, predisposing factors, compatible radiological findings, and histopathological and/or microbiological evidence of Mucorales which is highly dependent on the available techniques and trained personnel [ 1- 7 , 11 , 14 , 24-26 ]. 

In SM, a computed-tomography scan usually reveals mucosal thickening [ 7 , 27 ] which was present in all of our SM patients. Signs of more invasive disease may be absent; however, any evidence of bone erosion or suspicion of involvement of the eye or brain should prompt magnetic resonance imaging (MRI) [ 2 , 4 , 7 ]. Nasal endoscopy is recommended as a method for the evaluation of the extent of the disease to obtain mycological samples and also conduction of follow-ups [ 24 , 25 ]. 

Diagnostic confirmation usually requires invasive sampling of lesions for histopathology, culture, and PCR [ 2 , 4 , 5 , 7 , 11 , 16 ]. When a biopsy is not possible, other available specimens should be collected, such as sputum or bronchoalveolar lavage in PM [ 2 , 7 ]. Given the fragility of Mucorales and friability of the affected tissue, careful and prompt management of every sample is critical since damage can lead to negative cultures and/or unrecognizable structures on histopathological examination [ 2 ]. In this study, 12 out of 15 cases were confirmed, two of them only through necropsy. Moreover, the tissue samples ensured the diagnosis in 9 out of 11 patients from whom they were collected which strengthens the need to properly collect and process these specimens. It must also be noted that the culture was successful only once. 

Molecular methods still lack standardization and clinical validation; however, they have been increasingly supported due to their high sensitivity and specificity [ 2 , 4 , 5 , 7 , 11 ]. An in-house technique for the detection of Mucorales has been available in our institution since 2017. This technique was performed on five patients and ensured diagnosis in three of them; moreover, in two of them, it was the only positive result [ 28 ]. As these techniques become more reliable, we believe that an earlier and more accurate diagnosis will be possible which contributes to the improvement of the outcome of mucormycosis [ 26 , 29 ]. 

Despite the fact that the sensitivity of the cultures is suboptimal, it was worse in this study than reported in the literature (up to 79%) as was the sensitivity of PCR [ 6 , 7 ]. We believe that this was related to handling and processing constraints and also a significant delay in sampling after the beginning of the treatment. Importance of collecting several samples and sending them timely and appropriately for the examination cannot be overemphasized. Therefore, effective communication among ID specialists, surgeons, microbiologists, and anatomopathologists is of paramount importance. 

Early empirical antifungal treatment is essential to prevent disease progression and death [ 2 , 3 , 5 , 7 ]. However, a significant delay between the emergence of initial symptoms and clinical suspicion is still reported [ 4 , 5 ] similar to the present study. Nonspecific symptoms and initial antibiotic trials, among other factors, are probable contributing factors [ 5 , 30 ]. 

The AMB is one of the most consistently active medications against Mucorales, and liposomal-AMB (L-AMB) 5-10 mg/Kg/day is currently the first-line treatment with high doses recommended if there is CNS involvement [ 1-5 , 7 , 14 , 15 ]. Other approved options are posaconazole which is mostly supported for maintenance or salvage therapy and isavuconazole that is also used for induction [ 1-4 , 20 ]. The AMB was used in all of the treated patients in this study (from 2013 on, L-AMB was the first-line treatment). It is still considered as the preferred regimen due to its broader spectrum until confirmation of the diagnosis and the greater experience with this medication [ 1-4 , 7 ]. 

Azoles were used in eight out of nine patients who survived beyond the first month after diagnosis with good overall tolerability except for recurrent phlebitis in one patient on intravenous (IV) isavuconazole and severe nausea and vomit in another patient on oral posaconazole. Some of the most challenging aspects of azole management are medication interactions, interference with food, and therapeutic drug monitoring (TDM) [ 4 ]. Concerns about posaconazole oral suspension bioavailability led to the development of delayed-release tablets, which along with IV formulation, are now preferred [ 4 ]. Dosages between different posaconazole formulations are not directly interchangeable, and TDM with levels of ≥ 1 µg/mL, which may take several days, is strongly recommended [ 4 ]. 

One SM patient was responding to L-AMB; however, acute kidney injury and difficult-to-manage hypokalaemia prompted a switch to isavuconazole. After 20 days, the MRI showed disease progression, and a new switch to IV posaconazole was made followed by good clinical and radiologic responses, not requiring any other surgical procedure. Maintenance treatment with oral posaconazole in this patient required doubling the recommended daily dose to achieve therapeutic levels. 

Isavuconazole TDM is not recommended routinely [ 4 ] and was not available in our center; hence, we could only hypothesize that isavuconazole failed in this patient due to its subtherapeutic levels. Therefore, despite the fact that isavuconazole can be considered a first-line choice, a low threshold for imaging re-evaluation should be maintained and, in our opinion, TDM should be considered, especially if there is CNS involvement [ 4 , 5 ]. 

There is no data to support the standard combination treatment, and it does not warrant better outcome nor prevent surgical debridement; however, it can be considered while the diagnosis is not confirmed or as salvage therapy. Susceptibility to antifungals may differ between species since some have shown better response to combination treatment in vitro, such as Mucor irregularis; however, the clinical relevance of these findings remains unproven [ 31 ]. Combination treatment was unsuccessfully attempted in one patient with clinical deterioration on AMB [ 2 , 4 , 5 , 14 , 24 , 25 , 32-35 ]. 

Treatment duration is not determined; nevertheless, it usually requires weeks to months [ 3 , 4 , 7 ]. Control of the predisposing underlying condition (s), along with clinical and radiological resolution (or at least stability) are essential criteria for antifungal interruption [ 1-4 , 7 , 36 ]. In this series, surviving patients were treated for at least six months. It should also be noted that no relapse has been detected until now. 

In addition to AMB, surgery has been successful to decrease mortality rates, particularly in SM and PM cases. It may also be crucial for the reduction of the fungal inoculum since extensive thrombosis, tissue infarction, and necrosis prevent access of antifungals to the affected tissue [ 2-5 , 7 , 37 ]. Repeated and extensive interventions may be needed for source control [ 2 , 4 , 5 , 37 ] as was the case in four out of eight patients submitted to surgery, three of whom required extensive orbital exenteration. 

The HBO may improve neutrophil function and healing process, inhibit fungal growth, and promote AMB action; besides, although data are missing, it might be beneficial, particularly to SM and CM cases [ 2 , 3 , 5 ]. It was used in 6 out of 11 patients with SM and resulted in progressive improvement and no serious adverse events. 

The overall mortality rate in our series was 47% which is in line with those of other series(4647%) [ 6 , 12 , 23 ]. Disseminated infection and CNS, gastrointestinal, or pulmonary involvement are associated with worse outcomes, as are uncontrolled underlying diseases, hematologic malignancy, and transplantation [ 1 , 3 , 4 , 11 , 12 , 20 , 21 , 23 ]. In our small series, PM involvement produced the worse outcomes. 

The present study had some limitations since it was retrospective and based on clinical records from a long time ago. It must be noted that since then the knowledge, recommendations, and available diagnostic techniques or treatments (e.g., endoscopic surgery, PCR, L-AMB, and new azoles) have significantly evolved. Moreover, the diagnosis was not available to the species level, nor was antifungal susceptibility testing. Nonetheless, given the unlikely availability of data from randomized studies in the near future, every effort should be made to share information that contributes to the expansion of knowledge about the disease and its optimal management. 

This study aimed to present our experience with this demanding disease, share our cumulative knowledge, discuss some difficult cases, and emphasize the importance of having this diagnosis in mind. Moreover, it aimed to underline the importance of a rapid and comprehensive approach to treatment and pursuit of a diagnosis. 

## Conclusion

Many aspects still need to be optimized in the management of mucormycosis; for instance, diagnostic methods should become faster and more effective. Furthermore, broader access must be available to antifungal susceptibility profiles and TDM. However, the authors strongly believe that a closer collaboration among ID specialists, surgeons, radiologists, microbiologists, and anatomopathologists is key to the improvement of the diagnosis, treatment, and prognosis of such a challenging infection. 

## Acknowledgments

Not applicable.

## Authors’ contribution

B.P.L. and I.A. contributed equally to this manuscript as joint first authors. Conceptualization: B.P.L., I.A, A.C.C., L.S. Methodology: B.P.L., I.A., A.C.C. Investigation: B.P.L., I.A. Writing – Original Draft: B.P.L., I.A. Writing – Review & Editing: B.P.L., I.A., A.C.C., A.S., L.S. Supervision: L.S.

##  Conflicts of interest

The authors declare that there was no conflict of interest in this study.

##  Financial disclosure

There were no sources of funding for this study.
